# Use of sugammadex in parotid surgery: a case report

**DOI:** 10.1186/s13256-016-0972-x

**Published:** 2016-06-24

**Authors:** Mustapha Bensghir, Abdelghafour Elkoundi, Redouane Ahtil, Mohammed Meziane, Charki Haimeur

**Affiliations:** Department of Anesthesiology, Military Hospital Med V Rabat, Faculty of Medicine and Pharmacy of Rabat, University of Souissi-Med V, Rabat, Morocco

**Keywords:** Parotid surgery, Neuromuscular blocking agents, Intubation, Sugammadex, Intraoperative facial nerve monitoring, Case report

## Abstract

**Background:**

Parotid surgery is a common ear, nose, and throat procedure. Facial nerve paralysis is the main feared complication following this surgery. To avoid this paralysis, intraoperative facial nerve monitoring is often used, but neuromuscular blocking agents interfere with this technique. Therefore, the neuromuscular blocking agent used should have a short duration of muscle relaxation. With the discovery of sugammadex, a steroidal neuromuscular blocking agent has acquired the potential to be used in place of succinylcholine.

**Case presentation:**

A 41-year-old African woman was scheduled for a parotidectomy at our hospital. Rocuronium-induced neuromuscular block was reversed intraoperatively with sugammadex to facilitate identification of facial nerve function. The facial nerve was identified without incident, and surgical conditions were good for the removal of the tumor. During postoperative follow-up, no evidence of residual paralysis has been noted.

**Conclusions:**

In parotid surgery, the use of sugammadex allows free use of a steroidal neuromuscular blocking agent for intubation and thus intraoperative facial nerve monitoring can be done safely.

## Background

Parotid surgery is a common ear, nose, and throat procedure. Transient or permanent facial nerve paralysis during this operation is still the most feared complication [1].

Intraoperative use of nerve integrity monitors during parotid surgery has been advocated to reduce the incidence of facial nerve paralysis, but neuromuscular blocking agents (NMBAs) interfere with the use of this technique. Therefore, the NMBA used should induce a short duration of muscle relaxation. Previously, the choice was limited to succinylcholine, which has some undesirable side effects, such as the risk of anaphylaxis, increased serum potassium levels, and other cardiovascular complications [2, 3]. The use of an anesthetic protocol without muscle relaxants might be responsible for difficult intubation, hemodynamic changes, and laryngeal complications.

With the discovery of sugammadex, steroidal NMBA has acquired the potential to be used in place of succinylcholine in parotid surgery with intraoperative facial nerve monitoring (IFNM). The use of sugammadex could allow better intubating conditions with better intraoperative identification of facial nerves, especially where contraindications to succinylcholine such as hyperkalemia, exist. This is the second report in the literature of parotid surgery in which rocuronium was administered and later antagonized with sugammadex for IFNM.

## Case presentation

**Fig. 1:**
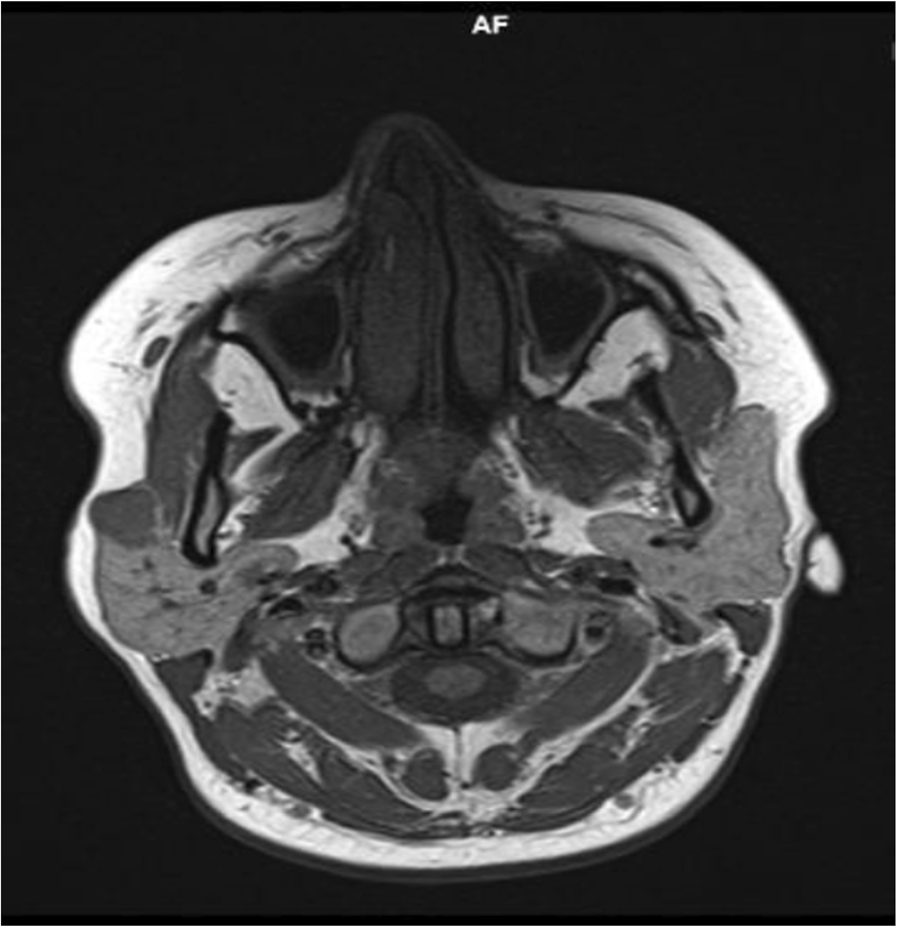
Axial T1-weighted image with hypointensity showing the tumor process of the right parotid gland

**Fig. 2:**
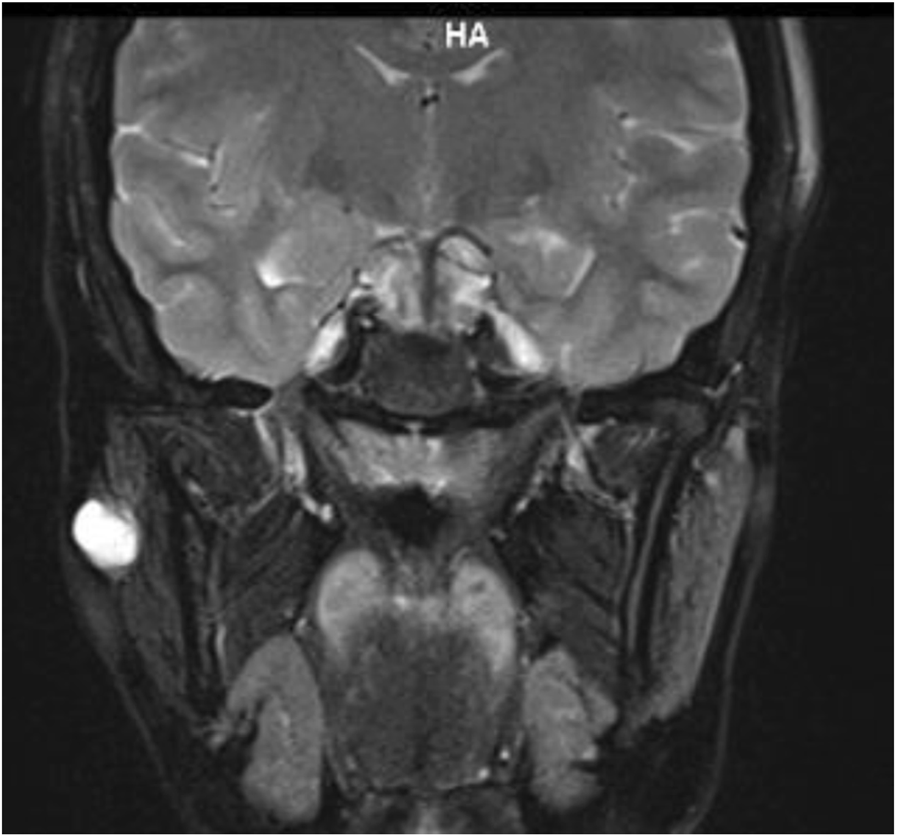
Coronal enhancement after gadolinium injection

A 41-year-old African woman weighing 72 kg and 169 cm tall, with American Society of Anesthesiologists physical status class I, was referred to our hospital because of tumefaction of the neck. Her clinical examination revealed an isolated parotid mass without associated lymphadenopathy. She had no pain or peripheral facial nerve palsy. The magnetic resonance imaging results were in favor of a pleomorphic adenoma (Figs. 1 and 2), and the patient was scheduled for a parotidectomy with general anesthesia.

The results of the patient’s cardiovascular examination were normal, with a noninvasive blood pressure of 131/71 mmHg and a heart rate of 79 beats/minute. Her respiratory examination revealed no dyspnea or snoring. Her oxygen saturation was 98 % on room air. An examination of her upper airway demonstrated good opening of the mouth and good mobility of the cervical spine (Mallampati class I). Her laboratory test results were a urea plasma concentration of 0.18 g/L, a creatinine level of 6 mg/L, blood glucose level of 0.99 mg/dl, hemoglobin concentration of 15.1 g/dl, platelet count of 213,000/mm^3^, prothrombin time of 12.9 seconds, and international normalized ratio of 1.2. Her chest x-ray and electrocardiogram (ECG) were unremarkable.

After written informed consent was obtained from the patient, it was decided to use general anesthesia during the procedure. Upon the patient’s arrival in the operating theater, intravenous access was established and standard anesthesia monitoring (three-lead ECG, peripheral oxygen saturation, noninvasive blood pressure) was instituted.

Neuromuscular monitoring was performed using acceleromyography. Following calibration, the ulnar nerve was supramaximally stimulated with a square pulse of 0.2-ms duration delivered as train-of-four (TOF) pulses at intervals of 15 seconds. The resulting contractions of the adductor pollicis muscles were quantified by using an acceleromyographic monitor (Infinity® Trident® NMT SmartPod®; Dräger, Lübeck, Germany). The TOF ratio and time interval from injection of the reversal agent to TOF ratio 0.9 were recorded.

Before induction of anesthesia, midazolam 2 mg was administered intravenously as premedication. After 5 minutes of adequate preoxygenation, anesthesia was initiated with fentanyl (2.5 μg/kg) and propofol (3 mg/kg) without significant hemodynamic changes. To facilitate tracheal intubation, rocuronium (0.5 mg/kg) was administered after effective mask ventilation. Once there was no twitching in response to TOF stimulation (1 minute, 45 seconds), the patient’s airway was successfully secured after the first attempt with a 7.0-mm endotracheal tube. The endotracheal tube was connected to a closed “low-flow” anesthetic breathing circuit. Ventilation was controlled to maintain normocapnia with a tidal volume of 8 ml/kg, a respiratory rate of 12 breaths/minute, and peak inspiratory pressure of 20 cmH_2_O. Anesthesia was maintained with isoflurane (1 %) in a mixture of oxygen and nitrous oxide (50 %:50 %). The end-tidal concentrations of anesthetic and carbon dioxide were measured continuously using a multiple gas monitor. The depth of anesthesia was monitored by bispectral index (BIS), and body temperature was maintained using heating blankets. The patient was placed in supine position and with slight Trendelenburg tilting of the table and the patient’s head turned to the left side. A bolus dose of fentanyl was administered before starting the skin incision to maintain a BIS score between 40 and 60. After the surgical incision was made (15 minutes after induction), there was no response on the basis of TOF ratio. Neuromuscular block was reversed with sugammadex (4 mg/kg). The TOF reached 0.9 after 5 minutes. No hemodynamic or respiratory changes were noted. We stopped the neuromuscular transmission (NMT) monitoring after obtaining a TOF ratio above 0.9 in three consecutive measurements.

The inspired isoflurane concentration was gradually increased to 1.8 % to prevent movement during critical phases of the surgical procedure after reversing neuromuscular blockade. The parotid gland was carefully dissected under magnification with facial nerve monitoring. The facial nerve was identified using the NIM 2.4 nerve integrity monitoring system (MEDTRONIC-XOMED, Jacksonville, FL, USA), and after 1 h surgical conditions were good for the removal of the tumor.

The patient was extubated when fully awake after a smooth emergence from anesthesia. Her oxygen saturation was 99 % with 2 L/minute of supplemental oxygen. Her postoperative course was uneventful, and no evidence of facial nerve paralysis was noted in the postinterventional surveillance room. The patient was discharged to home after 5 days of hospitalization.

## Discussion

Facial nerve palsy remains a serious complication after parotid surgery [1, 4]_._ It is feared by the surgical team and has a negative emotional and functional impact on the patient. The incidence of temporary facial nerve dysfunction may be quite high, with some authors reporting occurrence in up to 76 % of patients. Permanent facial nerve paralysis occurs much less frequently; some authors have reported rates of approximately 4–5 % [5, 6]. Risk factors for facial nerve injury include advanced age, inflammatory diseases of the parotid gland, duration of surgery, revision surgery, extent of surgery, and malignancy [5, 7–9].

Positive identification and preservation of the facial nerve is the key to preventing inadvertent facial nerve injury. IFNM has been advocated to reduce the incidence of facial nerve paralysis in parotid surgery. The technique is used for assessing the anatomic and functional integrity of nerves during parotid surgery and can be valuable in the presence of a high risk of nerve injury. This method requires intact NMT. However, the value of the procedure may be limited by the administration of NMBAs, which are required to prevent tracheal and/or laryngeal damage, difficult intubation, and hemodynamic changes during endotracheal intubation [10, 11]. To minimize the effect of this neuromuscular blockade, short-acting NMBAs such as succinylcholine and rocuronium were used. Succinylcholine has some undesirable side effects, such as the risk of anaphylaxis, increased serum potassium levels, and other cardiovascular complications [2, 3].

An alternative method is to use a nondepolarizing neuromuscular block; however, without reversal, waiting for a spontaneous recovery may be time-consuming and can potentially prolong the surgery. Before the introduction of sugammadex into clinical practice, neuromuscular block was routinely antagonized with neostigmine. This agent inactivates acetylcholinesterase, which is responsible for breakdown of acetylcholine and for displacing NMBAs to acetylcholine from the nicotinic receptors. Because NMBAs have a longer duration of action than cholinesterase inhibitors, there is a risk of recurarization. This explains why antagonism of intense blockade may be inefficient after neostigmine administration. Neostigmine is also associated with an increased risk for muscarinergic side effects, and parasympatholytic anticholinergic drugs such as atropine or glycopyrrolate need to be added. Another inconvenience is its substantial variability of action [12].

Sugammadex has been suggested as a superior alternative. It is a modified γ-cyclodextrin specifically developed for rapid reversal of a rocuronium- or vecuronium-induced neuromuscular blockade achieved by forming a stable and inactive complex and completely encapsulating these agents. Up to 90 % of the sugammadex dose is excreted within 24 h. Overall, 96 % of the dose is excreted in the urine, of which at least 95 % could be attributed to unchanged sugammadex. Administration of sugammadex to healthy volunteers resulted in increased renal elimination of rocuronium in complex with sugammadex. This results in reducing the amount of NMBAs available to bind to nicotinic receptors in the neuromuscular junction and in the reversal of neuromuscular blockade. This particular mechanism allows safe and effective antagonizing that can be applied at any time during the neuromuscular block by increasing doses of sugammadex. It is also devoid of the risks of neostigmine and has better efficacy at reversal. A phase III, active-controlled, randomized study proved that, compared with neostigmine, sugammadex provides significantly faster reversal of a deep rocuronium-induced neuromuscular blockade [13]. In our patient, sugammadex was administered at a TOF count of 0. The time to recovery of the TOF ratio to 0.9 was 5 minutes, which allowed IFNM to be conducted. Sugammadex was given in a 4 mg∙kg^−1^ dose. Fabregat *et al*. reported two patients [14] in whom the sugammadex dosage was seemingly lower than the one we used. Rocuronium-induced neuromuscular block was reversed with sugammadex 0.22 mg∙kg^−1^ when the TOF ratio was 0.14 in the first patient, and with sugammadex 2 mg∙kg^−1^ during intense block (posttetanic count = 0) in the second patient. The times to recovery of the TOF ratio to 0.9 were 5 minutes and 6 minutes, 15 seconds, respectively.

We chose to completely reverse neuromuscular block using sugammadex 4 mg∙kg^−1^ for different reasons. The use of IFNM alone does not guarantee preservation of facial nerve function. An important factor is the experience of the surgery team. The surgery was done by experienced surgeons who are known to reach the facial nerve very early with a significantly shorter interval between skin incision and facial nerve identification. This explains why sugammadex was administered earlier in the surgery at a deeper level of blockade. In addition, the nerve in such cases is chronically compressed by the tumor and may be more sensitive to the effects of neuromuscular blockade.

## Conclusions

Because of the importance of the facial nerve, its preservation is a central goal of parotid surgery. Rapid reversal of rocuronium-induced neuromuscular block by sugammadex, coupled with the analysis of neuromuscular blockade with the TOF method and IFNM, ensured the preservation of neuromuscular function and created optimal conditions for the surgical team in this case.

## Abbreviations

BIS, bispectral index; ECG, electrocardiogram; IFNM, intraoperative facial nerve monitoring; NMBA, neuromuscular blocking agent; NMT, neuromuscular transmission; TOF, train of four.
